# Identification of a Shrimp E3 Ubiquitin Ligase TRIM50-Like Involved in Restricting White Spot Syndrome Virus Proliferation by Its Mediated Autophagy and Ubiquitination

**DOI:** 10.3389/fimmu.2021.682562

**Published:** 2021-05-11

**Authors:** Chao Zhao, Chao Peng, Pengfei Wang, Lulu Yan, Sigang Fan, Lihua Qiu

**Affiliations:** ^1^ Key Laboratory of South China Sea Fishery Resources Exploitation and Utilization, Ministry of Agriculture and Rural Affairs, South China Sea Fisheries Research Institute, Chinese Academy of Fishery Sciences, Guangzhou, China; ^2^ Sanya Tropical Fisheries Research Institute, Sanya, China; ^3^ Key Laboratory of Aquatic Genomics, Ministry of Agriculture and Rural Affairs, Chinese Academy of Fishery Science, Beijing, China

**Keywords:** *Penaeus monodon*, PmTRIM50-like, WSSV, autophagy, ubiquitination

## Abstract

Most tripartite motif (TRIM) family proteins are critical components of the autophagy machinery and play important roles in host defense against viral pathogens in mammals. However, the roles of TRIM proteins in autophagy and viral infection have not been studied in lower invertebrates, especially crustaceans. In this study, we first identified a *TRIM50-like* gene from *Penaeus monodon* (designated *PmTRIM50-like*), which, after a white spot syndrome virus (WSSV) challenge, was significantly upregulated at the mRNA and protein levels in the intestine and hemocytes. Knockdown of *PmTRIM50-like* led to an increase in the WSSV quantity in shrimp, while its overexpression led to a decrease compared with the controls. Autophagy can be induced by WSSV or rapamycin challenge and has been shown to play a positive role in restricting WSSV replication in *P. monodon*. The mRNA and protein expression levels of PmTRIM50-like significantly increased with the enhancement of rapamycin-induced autophagy. The autophagy activity induced by WSSV or rapamycin challenge could be inhibited by silencing *PmTRIM50-like* in shrimp. Further studies showed that rapamycin failed to induce autophagy or inhibit WSSV replication after knockdown of *PmTRIM50-like*. Moreover, pull-down and *in vitro* ubiquitination assays demonstrated that PmTRIM50-like could interact with WSSV envelope proteins and target them for ubiquitination *in vitro*. Collectively, this study demonstrated that PmTRIM50-like is required for autophagy and is involved in restricting the proliferation of WSSV through its ubiquitination. This is the first study to report the role of a TRIM family protein in virus infection and host autophagy in crustaceans.

## Introduction

Protein post-translational modifications, such as phosphorylation, acetylation, and ubiquitination, are important biological processes that regulate many intracellular signaling pathways (1). Ubiquitination is a post-translational modification pathway that regulates many host cellular processes, including DNA repair, differentiation, and regulation of immune responses (2). Ubiquitination involves the covalent attachment of ubiquitin to a lysine residue on the substrate protein (3). The process of ubiquitination requires three enzymes: E1 ubiquitin-activating enzyme, E2 ubiquitin-conjugating enzyme, and E3 ubiquitin ligase to accomplish three distinct activities including activation, conjugation, and ligation, respectively (4). E3 ubiquitin ligases are mainly responsible for determining the substrate specificity (5). These enzymes operate in a concerted manner to poly-ubiquitinate a substrate protein and define its subsequent fate (6). The chain of ubiquitin molecules regulates molecular signaling depending on the lysine linkage type of the poly-ubiquitin moiety, its length, and additional post-translational modifications. Poly-ubiquitin chains can be formed *via* seven distinct lysine residues (K6, K11, K27, K29, K33, K48, and K63). The type of lysine linkage generally determines the fate of the substrate protein. For example, K48-linked chain-directed ubiquitination of substrate protein leads to its proteasomal degradation, whereas a substrate protein conjugated with K63-linked chain is targeted for kinase-mediated cell signaling (7).

Over 600 putative E3 ubiquitin ligases are encoded in the human genome (8). Based on domain structures, E3 ubiquitin ligases have been classified into two major families: homologous to the E6-AP COOH terminus (HECT) family and the RING-finger-containing protein family (9, 10). The tripartite motif (TRIM) proteins, which contain a typical RING-finger-containing domain, are a group of highly conserved proteins that participate in a variety of biological processes such as regulation of development, autophagy, apoptosis, carcinogenesis, and innate immunity (11). Because they contain a RING-finger domain, most TRIM proteins have been defined as E3 ubiquitin ligases and hence contain E3 ubiquitin ligase activity (11). TRIM proteins play an integral role in mammalian defense against pathogens. In vertebrates, TRIM proteins exert antiviral activity by at least three major mechanisms: directly antagonizing specific viral components, regulating transcription-dependent antiviral responses such as proinflammatory cytokine induction, and modulating other important cell-intrinsic defense pathways such as autophagy (12).

Autophagy is an evolutionarily conserved process that can lead to the formation of autophagic lysosomes to wrap and degrade damaged proteins or organelles and pathogen-derived components (13). Autophagy is induced by many different viruses, yet the impact of autophagy on viral replication is highly virus- and cell type-specific (14). Autophagy can serve as an antiviral defense pathway by directly targeting virus or its components for degradation (15, 16). Additionally, autophagy can activate innate and adaptive immunity by capturing and exposing viral components to specific pattern recognition receptors or antigen-presenting cells (17). For example, autophagy might be involved in delivering viral nucleic acids to Toll-like receptor 7 (TLR7), which mediates the induction of type 1 interferon (IFN) production. Moreover, autophagy might be involved in delivering endogenously synthesized microbial antigens and self-antigens to late endosomes, which are loaded onto MHC class II molecules for presentation to CD4+ T cells (16). However, autophagy could also dampen the antiviral defense pathway by degrading the signaling components of the cytokine response, thereby inhibiting antiviral gene expression (18). Recent studies have shown that TRIM proteins play an important role in regulating autophagy and viral infections (18). Using siRNA screening, over 30 TRIM proteins have been found to trigger autophagy induction (19). TRIM20 and TRIM21 are essential for IFNγ-induced autophagy (20). TRIM5a plays an important role in pp242 (an mTOR inhibitor)-induced autophagy (21). TRIM50 is a p62/SQSTM1 interacting protein that promotes the formation and autophagy clearance of aggresome-associated polyubiquitinated proteins through HDAC6 interaction (22, 23). TRIM23 mediates virus-induced autophagy *via* the activation of TANK-binding kinase 1 (TBK1) (24). The role of TRIM proteins in antiviral autophagy involves at least two different mechanisms: acting as specific cargo receptors that directly recognize viral components and target them for degradation (20, 25), or interacting with cargo/target-recognizing proteins such as p62 and core regulators of autophagy and forming protein complexes called ‘TRIMosomes’ (11). Although the role of TRIM proteins in regulating innate immunity and autophagy has been well established in mammals, reports on the role of TRIM proteins in aquatic invertebrates are very limited, except the report showing that TRIM9 homologs from *Litopenaeus vannamei* and the oyster *Crassostrea hongkongensis* can act as negative regulators of the NF-κB pathway (26, 27). There are still no reports about the role of TRIM proteins in autophagy and viral infection in crustaceans.

Black tiger shrimp (*Penaeus monodon*), which is one of the three major cultured shrimp species in the world, has important economic value (28). However, with the rapid development of intensive aquaculture and severe pollution of the marine ecological environment, the outbreak of diseases poses a serious threat to the shrimp farming industry, which greatly limits the sustainable development of shrimp aquaculture (29). White spot syndrome virus (WSSV) is one of the most virulent pathogens in shrimp leading to huge economic losses in the shrimp industry (30). Great progress has been made in the research on molecular immune mechanisms of shrimp against WSSV infection. For example, scavenger receptor C of *Marsupenaeus japonicus* interacted with WSSV VP19 *via* its extracellular domain and invoked hemocyte phagocytosis to restrict WSSV systemic infection (31). The polymeric immunoglobulin receptor of *Marsupenaeus japonicas* interacts with VP24 and mediates WSSV internalization *via* the pIgR-CaM-Clathrin endocytosis pathway to facilitate virus proliferation (32). However, no effective method has been established to restrict the uncontrolled occurrence and rapid spread of WSSV disease. Understanding the molecular immune mechanism against WSSV infection might help identify new strategies to prevent WSSV infection.

In this study, an E3 ubiquitin ligase, PmTRIM50-like, was identified in *P. monodon*, and the anti-WSSV function of PmTRIM50-like was analyzed during WSSV infection.

## Materials and Methods

### Experimental Animals and Sample Preparation

Healthy shrimp (*P. monodon*; mass 15 ± 2 g) were collected from the Zhuhai Experimental Base of the South China Sea Fisheries Research Institute, China Academy of Fisheries Sciences (Guangdong, China). The shrimp were acclimated for two days in aerated seawater (5% salinity) at 24 ± 1°C. Healthy tissues from three randomly selected shrimp were examined to determine the distribution of PmTRIM50-like in the muscle, hepatopancreas, intestine, heart, hemocytes, stomach, brain, and gills. The samples were snap frozen in liquid nitrogen and stored at 80°C until use.

### WSSV Challenge and Tissue Collection

The WSSV inoculum was extracted from WSSV-infected shrimp and viral quantification was detected by quantitative real-time PCR (qPCR) as described in a previous study (33). Each shrimp was injected with 100 μl of WSSV virions (1×10^6^ copies/mL). One hundred microliters of sterile phosphate-buffered saline (PBS) (140 mM NaCl, 2.7 mM KCl, 10 mM Na_2_HPO_4_, and 1.8 mM KH_2_PO_4_, pH 7.4) was injected into shrimp used as control. Intestines and hemocytes were collected from the shrimp at different time points (0, 3, 6, 12, 24, 48, and 72 h post injection) for RNA or protein extraction. For hemocyte collection, shrimp hemolymph was extracted using a 1 mL syringe preloaded with anticoagulant (0.45 M NaCl, 10 mM KCl, 10 mM EDTA, and 10 mM HEPES; pH 7.45). After centrifugation at 800 × g for 6 min at 4°C, hemocytes were collected and used to extract RNA or protein. At least three shrimp were used for tissue collection in each group.

### Rapamycin and Chloroquine Injection

Rapamycin (Rap) is widely used to induce autophagy by directly targeting rapamycin receptor (TOR) kinase (34, 35). Rapamycin (5 mg/kg) was used to induce autophagy in the Chinese mitten crab, *Eriocheir sinensis* (36). Chloroquine (CHQ) is a well-established inhibitor of autophagic proteolysis, which acts by inhibiting acidification of lysosomes and endosomes (37). Chloroquine (10 mg/kg) was used to inhibit lysosomal activity in shrimp (31). In this study, rapamycin was diluted to different concentrations (0.1, 0.5, 1, 2, and 4 mM) with PBS. Chloroquine was diluted to different concentrations (0.5, 1, 2, 4, and 8 mM) with PBS. Different concentrations of rapamycin or chloroquine (100 μl) were injected intramuscularly into virus-free shrimp. By evaluating the toxic effects of different concentrations of rapamycin or chloroquine on shrimp, we found that 4 mM rapamycin was toxic to shrimp (data not shown), whereas 1 mM rapamycin was not toxic but was effective in inducing autophagy. Although 8 mM chloroquine did not show toxicity to shrimp (data not shown), 4 mM chloroquine was sufficient to inhibit autophagy. Therefore, 1 mM rapamycin and 4 mM chloroquine were chosen to activate or inhibit shrimp autophagy activity in further experiments.

After treatment with PBS, WSSV, rapamycin, or chloroquine, shrimp hemocytes and intestines were collected at various time points after injection (0, 6, 12, 24, 48, and 72 h) to detect PmTRIM50-like expression or autophagy. To investigate the relationship between autophagy and WSSV infection, shrimp were first treated with PBS, rapamycin, or chloroquine, and then 12 h later, challenged with WSSV. The survival rate was recorded within 96 h of WSSV injection. Previous studies have shown that the expression level of *VP28* acts as a marker of WSSV replication, which indicates the number of virus replications (31). In this study, shrimp intestines and hemocytes were collected after 72 h of WSSV challenge to detect the expression levels of *VP28* and virus copies.

### Quantification of WSSV Copies

The open reading frame (ORF) of the WSSV *VP28* fragment was amplified and inserted into the pMD18-Tvector (TaKaRa, Dalian, China). The recombinant plasmid was quantified using a NanoDrop 2000/2000C (Thermo Scientific, Beijing, China). The copy number of the plasmid was calculated using the known molecular weight of the plasmid. Subsequently, the plasmid was diluted gradually (10^9^, 10^8^, 10^7^, 10^6^, 10^5^, 10^4^, 10^3^) and the diluted samples were used as templates for qPCR with primers VP28-RT-F and VP28-RT-R (**Table 1**). The cycle threshold (CT) values and the quantity of the template were used to generate a standard curve for WSSV quantification. Genomic DNA extracted from the viral inoculum or WSSV-infected tissue, along with the gradient diluted plasmid samples, was analyzed by qPCR to obtain the absolute copies of WSSV from the inocula or infected tissue.

**Table 1:** Primers used in this study.

Primers	Sequence (5’→3’)	Usage
TRIM50-like-F	ATGCTGCCTGAGTGCGGTC	ORF cloning
TRIM50-like-R	TCAATTCGTCGCAAAGTCGC	ORF cloning
rTRIM50-like-F	TACTCAGAATTCATGCTGCCTGAGTGCGGTC	Recombinant expression
rTRIM50-like-R	TACTCACTCGAGTCAATTCGTCGCAAAGTCGC	Recombinant expression
rVP19-F	GAATTCATGGCCACCACGACTAACAC	Recombinant expression
rVP19-R	CTCGAGTTAATCCCTGGTCCTGTTCTTAT	Recombinant expression
rVP24-F	GAATTCATGCACATGTGGGGGGTTTA	Recombinant expression
rVP24-R	CTCGAGTTATTTTTCCCCAACCTTAA	Recombinant expression
rVP26-F	GGATCCACACGTGTTGGAAGAAGCGT	Recombinant expression
rVP26-R	GAATTCTTACTTCTTCTTGATTTCGTCCTTG	Recombinant expression
rVP28-F	GAATTCATGGATCTTTCTTTCACTCTTTCGG	Recombinant expression
rVP28-R	CTCGAGTTACTCGGTCTCAGTGCCAGAGTAG	Recombinant expression
VP28-RT-F	CTCCGCAATGGAAAGTCTGA	qRT-PCR
VP28-RT-R	GGGTGAAGGAGGAGGTGTT	qRT-PCR
β-actin-F	CCCTGTTCCAGCCCTCATT	qRT-PCR
β-actin-R	GGATGTCCACGTCGCACTT	qRT-PCR
qTRIM50-like-F	AGCGCTAGGGGAGTGTCATA	qRT-PCR
qTRIM50-like-R	CACAATGGTCACGTCCCTCA	qRT-PCR
dsTRIM50-like-F	GATCACTAATACGACTCACTATAGGGCCTTCTGCAGGGAGTGTCTC	RNA interference
dsTRIM50-like-R	GATCACTAATACGACTCACTATAGGGCACACCGCAGCTTCATCTTA	RNA interference
dsGFP-F	CGAGCTCTGGAGTGGTCCCAGTTCTTGTTGA	RNA interference
dsGFP-R	ACGCGTCGACGCCATTCTTTGGTTTGTCTCCCAT	RNA interference

### RNA Extraction, cDNA Synthesis, and DNA and Protein Extraction

Total RNA was extracted from approximately 50 mg of tissue obtained from shrimp using TRIzol reagent (Invitrogen, Shanghai, China) according to the manufacturer’s instructions. After detecting the concentration and quality of total RNA, the PrimeScript reverse transcriptase kit (TaKaRa, Dalian, China) was used for reverse transcription with 1 μg of total RNA according to the manufacturer’s instructions. The RNA extract and cDNA were stored at −80°C until use. Genomic DNA was extracted using a genomic DNA extraction kit (Tiangen, Beijing, China). Protein samples from different organs were homogenized separately in radioimmunoprecipitation assay (RIPA) buffer (50 mM Tris-HCl, 150 mM NaCl, 0.1% SDS, 0.5% Nonidet P-40, 1 mM EDTA, 0.5 mM PMSF, pH 7.5). The tissue homogenate was centrifuged at 12000 × g for 10 min at 4°C to collect the supernatant for further analysis.

### cDNA Cloning and Sequence Analysis

The ORF sequence was amplified using PmTRIM50-like-F/R primers (**Table 1**). The PCR program for amplification was one cycle of 94°C for 3 min; 40 cycles of 94°C for 30 s, 60.5°C for 30 s, and 72°C for 1.5 min, followed by one cycle of 72°C for 10 min. The resulting PCR product was further verified by electrophoresis on a 1.2% agarose gel and purified by PCR. The product was ligated into the pMD18-Tvector (TaKaRa, Dalian, China) and transformed into DH5α cells, and the positive clones were picked for sequencing (Ruibiotech, China).

The *PmTRIM50-like* sequence was analyzed using the BLAST program (http://blast.ncbi.nlm.nih.gov/Blast.cgi) from the NCBI server. Protein domain analysis of PmTRIM50-like was performed using the conserved domain search service (https://www.ncbi.nlm.nih.gov/Structure/cdd/wrpsb.cgi). The molecular size and theoretical isoelectric point of PmTRIM50-like were analyzed online using ExPASy software (http://www.expasy.org/). Phylogenic analysis was performed using the neighbor-joining (NJ) method using ClustalW and MEGA 6. All PmTRIM50-like alignment sequences were from the GenBank database (XP_037788838.1, E3 ubiquitin-protein ligase TRIM11-like [*Penaeus monodon*]; MPC09098.1, Tripartite motif-containing protein 10 [*Portunus trituberculatus*]; MPC27339.1, Tripartite motif-containing protein 10 [*Portunus trituberculatus*]; MPC09100.1, Tripartite motif-containing protein 72 *[Portunus trituberculatus*]; KAB7494620.1, E3 ubiquitin-protein ligase TRIM50 [*Armadillidium nasatum*]; ACU46018.1, TRIM5/cyclophilin A fusion protein [*Macaca fascicularis*]; KFU97184.1, Tripartite motif-containing protein 59, partial [*Pterocles gutturalis*]; XP_006123500.1, tripartite motif-containing protein 59 [*Pelodiscus sinensis*]; XP_007441599.1, E3 ubiquitin-protein ligase TRIM50 [*Python bivittatus*]; XP_012302766.1, tripartite motif-containing protein 59 [*Aotus nancymaae*]; XP_003256425.1, tripartite motif-containing protein 59 [*Nomascus leucogenys*]; XP_017949750.1, E3 ubiquitin-protein ligase TRIM50 [*Xenopus tropicalis*]; XP_020844498.1, tripartite motif-containing protein 59 [*Phascolarctos cinereus*]; XP_017265762.1, E3 ubiquitin-protein ligase TRIM13 [*Kryptolebias marmoratus*]; EMP32723.1, Tripartite motif-containing protein 59, partial [*Chelonia mydas*]; XP_032071003.1, E3 ubiquitin-protein ligase TRIM50 [*Thamnophis elegans*]; KGL84188.1, Tripartite motif-containing protein 59, partial [*Tinamus guttatus*]; XP_037362732.1, E3 ubiquitin-protein ligase TRIM50 [*Talpa occidentalis*]; and NP_775107.1, tripartite motif-containing protein 59 [*Homo sapiens*]).

### Recombinant Expression, Purification, and Antiserum Production of PmTRIM50-Like

The ORF of *PmTRIM50-like* was amplified using the PCR primers PmTRIM50-like-F and PmTRIM50-like-R (**Table 1**) and cloned into the pET32a plasmid. The recombinant plasmid was transformed into *E. coli* BL21 cells, and the protein was induced with 0.6 mM isopropyl-β-D-thiogalactopyranoside when the OD_600_ of the bacterial concentration reached 0.6. Recombinant rPmTRIM50-like was purified by affinity chromatography using a His-bind purification kit (Beyotime, Shanghai, China). The protein was separated by 12% sodium dodecyl sulfate-polyacrylamide gel electrophoresis (SDS-PAGE) and visualized using a GE Image scanner III. Rabbit antiserum against PmTRIM50-like was prepared following a previously reported method (38).

### Western Blotting Analysis

The purified protein or total protein extracted from shrimp tissue was separated by SDS-PAGE and transferred onto nitrocellulose membranes. The membranes were blocked in 5% skim milk and resuspended in 0.1 M phosphate buffer solution-Tween (PBST) at room temperature for 1 h. The membranes were washed with 0.1 M PBST three times for five min each. To detect the purified PmTRIM50-like protein, the membranes were incubated with 6×His-Tag HRP Antibody (SAB, Guangzhou, China; 1:3000 in PBS) or HRP-conjugated GST tag mouse monoclonal antibody (Proteintech, Guangzhou, China; 1:20000 in PBS) for 1–2 h at 37°C per the manufacturer’s instructions, and washed with 0.1 M PBST three times for ten min each. For detecting the endogenous proteins, the membranes were incubated with antiserum against PmTRIM50-like (1:500 dilution in 0.1 M PBST), anti-LC3 antibody (Abcam, Shanghai, China; 1:2000 dilution in 0.1 M PBS), anti-VP28 antibody (Abcam, Shanghai, China; 1:500 dilution in 0.1 M PBS), or anti-β-actin antibody (SAB, Guangzhou, China; 1:500 dilution in 0.1 M PBS) for 2 h at room temperature. The membranes were washed three times for ten min with 0.1 M PBST and then incubated with horseradish peroxidase (HRP)-conjugated goat anti-rabbit antibodies (SAB, Guangzhou, China; 1:20000 dilution in 0.1 M PBS). Immune active bands were visualized by staining with horseradish peroxidase (HRP)- diaminobenzidine (Tiangen, Beijing, China) and visualized with a GE Image scanner III. β-actin expression was used to normalize the amount of loaded proteins.

To detect the occurrence of autophagy in shrimp, the protein expression level of LC3-II/LC3-I was adopted as the reference standard in the present study. The ratio of LC3-II/LC3-I has been proven to be a hallmark of autophagy. LC3 was transformed from LC3-I to LC3-II upon induction of autophagy, and the electrophoresis rate of lipid-acylated LC3-II in polyacrylamide gel was faster than that of LC3-I (39).

### Quantitative Real Time PCR

The mRNA expressions of *PmTRIM50-like* and *VP28* were detected by qRT-PCR with *β-actin* (GenBank accession No. JN808449.1) used as an internal control. The primers used for qRT-PCR are listed in **Table 1**. qRT-PCR was performed in triplicate for each concentration using 384-well plates with a total volume of 12.5 μL, including 6.25 μL of 2×SYBR Preix Ex Taq II (TaKaRa, Dalian, China), 0.5 μL of each specific primer, 1 μL of the cDNA template, and 4.25 μL of DEPC water. The program was as follows: denaturation at 94°C for 2 min; 40 cycles of 94°C for 15 s, 58°C for 20 s, and 72°C for 20 s. The PCR product was denatured to produce a melting curve to determine its specificity. Each reaction was carried out in three separate tubes, and the test was repeated three times. PCR data were calculated using the 2^−ΔΔCT^ method and expressed as the mean ± SD. Student’s *t*-test was used to analyze the significant differences among PCR data, and a significant difference was accepted at *p < 0.05*.

### RNA Interference and Overexpression

Primers *dsPmTRIM50*-*like*-F and *dsPmTRIM50*-*like*-R (**Table 1**), incorporating a T7 promoter, were used to amplify the template to produce *dsRNA* with T7 RNA polymerase. Primers of *dsGFP*-F and *dsGFP*-R with the T7 promoter sequences (**Table 1**) were used to clone a 289 bp DNA fragment of the green fluorescent protein (*GFP*) gene. *dsRNA* was synthesized *in vitro* using the Transcription T7 Kit (TaKaRa, Dalian, China) following the manufacturer’s protocol. *dsRNA* (50 μg) of *dsPmTRIM50-like* was injected into the abdominal segment of each shrimp. *dsGFP* was used as a control. The intestine and hemocytes were collected at 24 h and 48 h post injection, and RNA interference efficiency at the mRNA and protein levels was detected by qRT-PCR and western blotting, respectively. *β-Actin* was used as an internal reference.

To investigate the role of PmTRIM50-like in autophagy or WSSV infection, the shrimp were first injected with *dsRNA* and then challenged with WSSV or Rap 12 h later. Shrimp intestines were collected for detecting autophagy after 12 and 24 h of WSSV or Rap challenge. Shrimp intestines and hemocytes were collected to detect virus replication after 72 h of WSSV challenge. The survival rates in the *dsPmTRIM50-like*+WSSV and *dsGFP*+WSSV groups were recorded within 96 h of WSSV injection. To investigate the role of PmTRIM50-like in autophagy and WSSV infection, the shrimp were first co-injected with *dsRNA* and Rap, and 12 h later, shrimp were challenged with WSSV. Shrimp intestines were collected at 12 h and 72 h post WSSV injection to detect autophagy or viral replication. The survival rates in the *dsPmTRIM50-like+*Rap+WSSV and *dsGFP*+Rap+WSSV groups were recorded within 96 h of WSSV injection.

Overexpression of the target gene was carried out by injecting mature body mRNA (31, 32). The recombinant pET32a-PmTRIM50-like plasmid was used as a template to transcribe single-stranded and capped *PmTRIM50-like* mRNAs using an mMESSAGE mMACHINE™ T7 Transcription Kit (Ambion Europe LTD, Cambridgeshire, UK), according to the manufacturer’s instructions. The empty pET32a vector was used as a template to synthesize Trx (thioredoxin)-His tag mRNA as a control. Each shrimp was injected with 100 μg *PmTRIM50-like* (or *Trx-His tag*) mRNA, and at least three shrimp were used in each group. After 24 h and 48 h of mRNA injection, the efficiency of overexpression at the mRNA and protein levels was detected by qRT-PCR and western blotting, respectively. *β-Actin* was used as an internal reference.

To investigate the effect of PmTRIM50-like overexpression on WSSV infection, the shrimp were first injected with *PmTRIM50-like* mRNA or *Trx-His tag* mRNA, and then challenged with WSSV 12 h later. Shrimp intestines and hemocytes were collected to detect virus replication after 72 h of WSSV challenge.

### Transmission Electron Microscopy

The intestine and hemocytes of WSSV-treated, Rap-treated, and PBS-treated shrimp were harvested at 12 h post injection. Tissues were fixed in 2.5% glutaraldehyde overnight at 4°C. The samples were post-fixed in 1% osmium tetroxide for 1 h, dehydrated with graded ethanol, and embedded in epoxy resin. Ultrathin sections were double-stained with uranyl acetate and lead citrate. Images were collected using JEM1400.

### Immunocytochemistry Assay

Intestines from the WSSV, Rap, PBS, *dsPmTRIM50-like*+WSSV, *dsGFP*+WSSV, *dsPmTRIM50-like*+Rap, and *dsGFP*+Rap groups were dissected after 12 h of WSSV or Rap challenge. Tissues were fixed with 4% paraformaldehyde diluted in PBS. Immunocytochemistry assays were performed following the method described by Zhang et al. (40). Briefly, after dehydration with ethanol, the tissues were embedded in paraffin, sectioned at a thickness of 5 μm, and rehydrated in water. The sections were blocked with 5% skim milk in PBS and then incubated with Anti-LC3 antibody (1:200 in 3% BSA; Abcam, Shanghai, China) for 1–2 h at 37°C. Antibody binding was visualized by fluorescence microscopy using FITC-conjugated goat anti-rabbit IgG (1:10,000 in PBS). DAPI (1:10,000 in PBS) was used to dye the nuclei for 10 min.

### Pull-Down Assay

Pull-down assays were performed to explore whether recombinant rPmTRIM50-like could interact with the main envelope proteins of WSSV (VP19, VP24, VP26, and VP28). The primers used for the recombinant expression of VPs (VP-F and VP-R) are shown in **Table 1**. The amplified sequences of VP19 (GenBank accession No. DQ681071.1), VP24 (GenBank accession No. DQ196431.1), VP26 (GenBank accession No. AY220746.1), and VP28 (GenBank accession No. DQ681069.1) were ligated separately into the vector PGEX-4T-1 and transformed into *E. coli* BL21 cells for expression. The recombinant proteins were purified by affinity chromatography using a GST-resin (Beyotime, Shanghai, China). For the pull-down assay, purified His-tagged rPmTRIM50-like was incubated with Ni-NTA beads, to which purified VP19, VP24, VP26, or VP28 was added and incubated at 4°C overnight with slight rotation. The mixture was washed thoroughly with wash buffer (20 nM imidazole, 50 mM Tris-HCl, pH 8.0), eluted in elution buffer (250 nM imidazole, 50 mM Tris-HCl, pH 8.0), and then analyzed using 12.5% SDS-PAGE, followed by western blotting with anti-GST antibody. For the GST pull-down assay, purified GST-tagged VP19, VP24, VP26, or VP28 and purified His-tagged rPmTRIM50-like were incubated with glutathione beads at 4°C overnight with slight rotation. The beads were washed with TBS thoroughly, the proteins were eluted with elution buffer (10 mM reduced glutathione and 50 mM Tris-HCl, pH 8.0), and then analyzed using 12.5% SDS-PAGE, followed by western blotting with anti-His antibody.

### *In Vivo* Ubiquitination Assay

The ubiquitination assay was performed as described by Choo et al. (41). The reaction mixture (40 μl) contained 8 μl of 5X ubiquitination buffer (100 mM Tris-HCl, pH 7.5, 25 mM MgCl_2_, 2.5 mM DTT, 10 mM ATP), 250 ng of ubiquitination E1 (E-305, Boston Biochem, Inc), 500 ng of ubiquitination E2 (E2-656, Boston Biochem, Inc), 500 ng of ubiquitin (U-110, Boston Biochem, Inc), 500 ng of rPmTRIM50-like, 250 ng of VP19, VP24, VP26, or VP28. The mixture was incubated at 37°C for 3 h. The reaction was stopped by adding SDS-PAGE sample buffer and boiling for 10 min. Ubiquitination was detected by western blotting with anti-Ub (Boston Biochem, Inc.; 1:1000 in PBS) and HRP-conjugated goat anti-rabbit IgG (1:20000). The VP19, VP24, VP26, and VP28 proteins were detected by western blotting using HRP-conjugated GST tag mouse monoclonal antibody (Proteintech, Guangzhou, China; 1:20000 in PBS).

## Results

### PmTRIM50-Like Was Upregulated by WSSV Challenge in Shrimp

The transcript of *PmTRIM50-like* obtained from the transcriptome database of *P. monodon* was validated by PCR and confirmed by sequencing. The full-length ORF of *PmTRIM50-Like* was 1245 bp, encoding a protein of 414 aa length (GenBank accession No. MW208625). The deduced amino acid sequence of PmTRIM50-like contained a typical RING domain of the RING-finger-containing protein family (positions 2–28 aa), a B-box domain (positions 64–106 aa), and a cyclophilin domain (positions 362–404 aa) (**Figure S1A**). To identify the phylogenetic relationships of PmTRIM50-like, a phylogenetic tree was constructed using the neighbor-joining method. The results showed that PmTRIM50-like was most closely related to the E3 ubiquitin-protein ligase TRIM50 of *Talpa occidentalis*, which formed a clade (**Figure S1B**).

**Figure 1:**
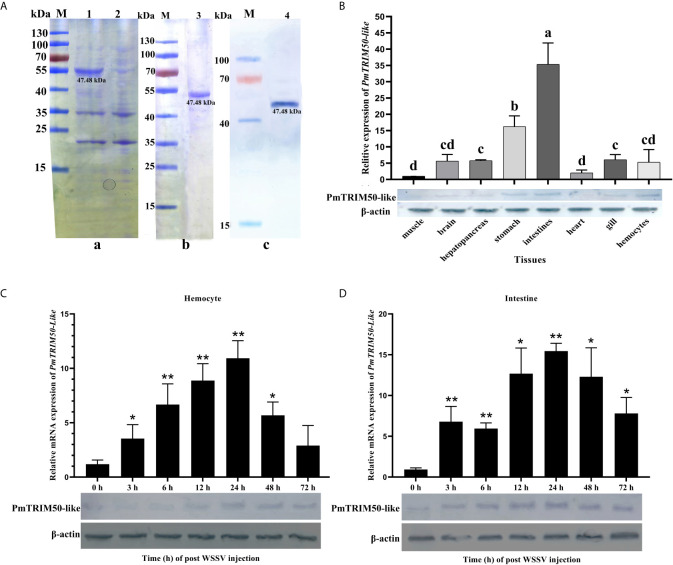
PmTRIM50-like was upregulated in shrimp by WSSV infection. **(A)** Expression of recombinant PmTRIM50-like in *E. coli* and protein purification: **(a)** SDS-PAGE of induced PmTRIM50-like; Lane M, molecular weight marker; 1, recombinant protein of PmTRIM50-like at 12-h-induced; 2, recombinant PmTRIM50-like protein of none-induced. **(b)** SDS-PAGE analysis of purified PmTRIM50-like; Lanes M, molecular weight marker; 3, purified PmTRIM50-like protein. **(c)** Western blotting of PmTRIM50-like; Lane M, molecular weight marker; Lane 4, 12-h-induced PmTRIM50-like protein. **(B)** Tissue distribution of PmTRIM50-like was analyzed by qRT-PCR (upper panel) and western blotting (lower panel). Different lowercase letters indicate statistically significant differences (*P < 0.05*). **(C, D)** Expression patterns of PmTRIM50-like mRNA and protein were detected by qRT-PCR (upper panel) and western blotting (lower panel) in hemocyte **(C)** and intestine **(D)** after WSSV challenge. *β-actin* was used as an internal reference. Asterisks indicate significant differences (*P< 0.05 and **P <0.01) compared with values in the control group. Error bars represent mean ± S.D. of three independent PCR amplifications and quantifications.

The recombinant PmTRIM50-like protein was then expressed and purified (**Figure 1A**). The theoretical molecular weight of the recombinant protein was 46.64 kDa, which consisted of a His tag (0.84 kDa) at the carboxyl terminus. The target protein (approximately 47.48 kDa) was detected in the supernatant of the resuspended bacteria that were destroyed by ultrasonication (**Figure 1Aa**); it was further confirmed by western blot analysis (**Figure 1Ac**). The recombinant protein was purified and stored at −80°C for use in subsequent experiments (**Figure 1Ab**). Anti-PmTRIM50-like sera were prepared using the purified recombinant protein PmTRIM50-like.

The spatial expression pattern of *PmTRIM50-Like* mRNA was determined by quantitative real-time PCR (qRT-PCR) using *β-actin* as an internal control. The results showed that *PmTRIM50-Like* mRNA was ubiquitously expressed in the intestine, stomach, hemocytes, gills, heart, brain, hepatopancreas, and muscle (**Figure 1B**, upper panel). Western blotting analysis showed that PmTRIM50-Like protein was also distributed in the intestine and hemocytes (**Figure 1B**, lower panel).

After challenge with WSSV, the expression of *PmTRIM50-like* mRNA in hemocytes increased significantly during 3–48 h post-challenge and then recovered to the same level as that in the PBS group. The peak value reached at 24 h post WSSV challenge, which was 10.6-fold higher than that of the control group (*p<0.01*, **Figure 1C**, upper panel). The expression of PmTRIM50-like protein in hemocytes began to increase at 12 h post WSSV challenge, as detected by western blotting (**Figure 1C**, lower panel). In the intestine, the expression of *PmTRIM50-Like* mRNA increased significantly during 3–72 h post challenge. The peak value also reached at 24 h post WSSV challenge (*p<0.01*, **Figure 1D**, upper panel), which was 16.6-fold higher than that of the control group (*p< < 0.01*, **Figure 1D**, upper panel). Western blotting results showed that the expression of PmTRIM50-like protein in the intestine began to increase at 3 h post WSSV challenge and was maintained at a high expression level for 6–72 h after challenge (**Figure 1D**, lower panel). These results suggest that PmTRIM50-like might be involved in WSSV infection in shrimp.

### PmTRIM50-Like Restricted WSSV Infection in *P. Monodon*

**Figure 2:**
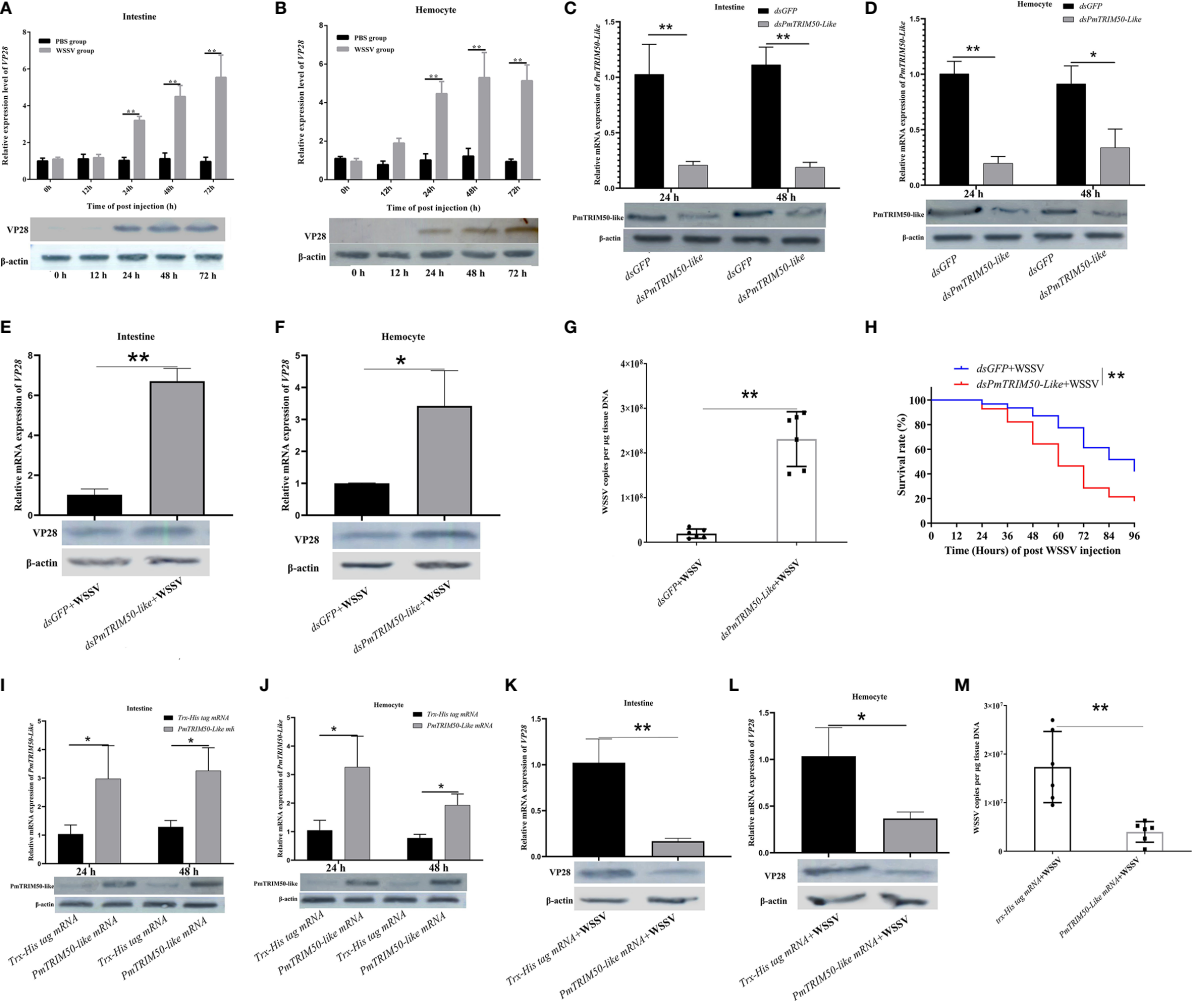
PmTRIM50-like restricted WSSV infection in shrimp. **(A, B)** Expression patterns of VP28 mRNA and protein were detected by qRT-PCR (upper panel) and western blotting (lower panel) in intestine **(A)** and hemocyte **(B)** after WSSV challenge. **(C)** Efficiency of *dsPmTRIM50-like* was detected by qRT-PCR (upper panel) and western blotting (lower panel) in the intestine. **(D)** Efficiency of *dsPmTRIM50-like* was detected by qRT-PCR (upper panel) and western blotting (lower panel) in hemocytes. **(E)** Expression of VP28 in the intestine of *dsPmTRIM50-like*+WSSV group and *dsGFP*+WSSV group was determined by qRT-PCR (upper panel) and western blotting (lower panel). **(F)** Expression of VP28 in hemocytes of *dsPmTRIM50-like*+WSSV group and *dsGFP*+WSSV group was determined by qRT-PCR (upper panel) and western blotting (lower panel). **(G)** The copy number of WSSV in the intestine of *dsPmTRIM50-like*+WSSV group and *dsGFP*+WSSV group was detected by qPCR. **(H)** The survival rate in *dsPmTRIM50-like*+WSSV group and *dsGFP*+WSSV group was detected after challenged by WSSV. Survival rates were analyzed statistically using the Kaplan-Meier plots (log-rank X2). **(I)** Efficiency of *PmTRIM50-like* overexpression was detected by qRT-PCR (upper panel) and western blotting (lower panel) in the intestine. **(J)** Efficiency of *PmTRIM50-like* overexpression was detected by qRT-PCR (upper panel) and western blotting (lower panel) in hemocyte. **(K)** Expression of VP28 in the intestine of *PmTRIM50-like* mRNA+WSSV group and *Trx-His tag* mRNA+WSSV group was determined by qRT-PCR (upper panel) and western blotting (lower panel). **(L)** Expression of VP28 in hemocytes of *PmTRIM50-like* mRNA+WSSV group and *Trx-His tag* mRNA+WSSV group was determined by qRT-PCR (upper panel) and western blotting (lower panel). **(M)** The copy number of WSSV in the intestine of *PmTRIM50-like* mRNA+WSSV group and *Trx-His tag* mRNA+WSSV group was detected by qPCR. The experiments were repeated three times. *β-actin* was used as an internal reference. Asterisks indicate significant differences *P< 0.05 and **P <0.01).

The replication of WSSV was detected using VP28 marker as shown in a previous study (31). In this study, when shrimp were challenged with WSSV, the expression level of WSSV *VP28* mRNA in the intestine increased significantly during 24–72 h compared to that of the PBS group (*p< < 0.01*, **Figure 2A**, upper panel). The expression of VP28 protein in the intestine began to increase at 24 h post WSSV challenge, as detected by western blotting (**Figure 2A**, lower panel). In hemocytes, the expression of *VP28* mRNA increased significantly during 24-72 h post challenge (*p< < 0.01*, **Figure 2B**, upper panel). Western blotting results showed that the expression of VP28 protein in hemocytes began to increase at 24 h post WSSV challenge (**Figure 2B**, lower panel). Hence, the expression of VP28 was used to detect WSSV replication in subsequent experiments.

To investigate the function of PmTRIM50-like in shrimp during WSSV challenge, RNA interference (RNAi), and mRNA overexpression of *PmTRIM50-like* were conducted. The results showed that the mRNA and protein expression levels of PmTRIM50-like decreased significantly in the intestine (**Figure 2C**) and hemocytes (**Figure 2D**) after 24 h and 48 h of *dsPmTRIM50-like* injection, indicating that PmTRIM50-like was successfully silenced by *dsPmTRIM50-like* treatment. The shrimp were treated with *dsGFP* or *dsPmTRIM50-like* and then challenged with WSSV. In the intestine and hemocytes, the expression of *VP28* mRNA was significantly increased in the *dsPmTRIM50-like*+WSSV group, which was 6.5-fold and 3.42-fold higher than that in the *dsGFP*+WSSV group, respectively (*p<0.01*, **Figure 2E**, upper panel and *p<0.05*, **Figure 2F**, upper panel). The expression of VP28 protein was also increased in the *dsPmTRIM50-like*+WSSV group compared to that in the *dsGFP*+WSSV group (**Figure 2E**, lower panel and **Figure 2F**, lower panel). In the intestine, the copy number of WSSV in the *dsPmTRIM50-like*+WSSV group (2.3 × 10^8^ copies per μg genomic DNA) was significantly higher than in the *dsGFP*+WSSV group (1.9 × 10^7^ copies per μg genomic DNA) (*p<0.01*, **Figure 2G**). In addition, the survival rate in the *dsPmTRIM50-Like*+WSSV group was 17.8%, which was significantly lower than that in the *dsGFP*+WSSV group (survival rate 41.9%) (*p< < 0.01*, **Figure 2H**).

The overexpression results indicated that PmTRIM50-like was successfully overexpressed at both mRNA and protein levels in the intestine and hemocytes of shrimp at 24 h and 48 h after injection of the *PmTRIM50-like* mRNA (**Figures 2I, J**). The shrimp were treated with *Trx-His tag* mRNA or *PmTRIM50-like* mRNA, followed by WSSV challenge. In the intestine and hemocytes, the expression of *VP28* mRNA was significantly decreased in the *PmTRIM50-like* mRNA+WSSV group, which was 0.16-fold and 0.35-fold higher than that in the *Trx-His tag* mRNA+WSSV group, respectively (*p<0.01*, **Figure 2K**, upper panel and *p<0.05*, **Figure 2L**, upper panel). Meanwhile, the expression of VP28 protein was decreased in the *PmTRIM50-Like* mRNA+WSSV group compared with the *Trx-His tag* mRNA+WSSV group (**Figure 2K**, lower panel and **Figure 2L**, lower panel). In the intestine, the copy number of WSSV in the *PmTRIM50-Like* mRNA+WSSV group (3.9 × 10^6^ copies per μg genomic DNA) was significantly lower than that in the *Trx-His tag* mRNA+WSSV group (1.7 × 10^7^ copies per μg genomic DNA) (*p<0.01*, **Figure 2M**). These results suggest that PmTRIM50-like restricts the replication of WSSV in shrimp.

### Autophagy Played a Positive Role in Restricting WSSV Infection in Shrimp

**Figure 3:**
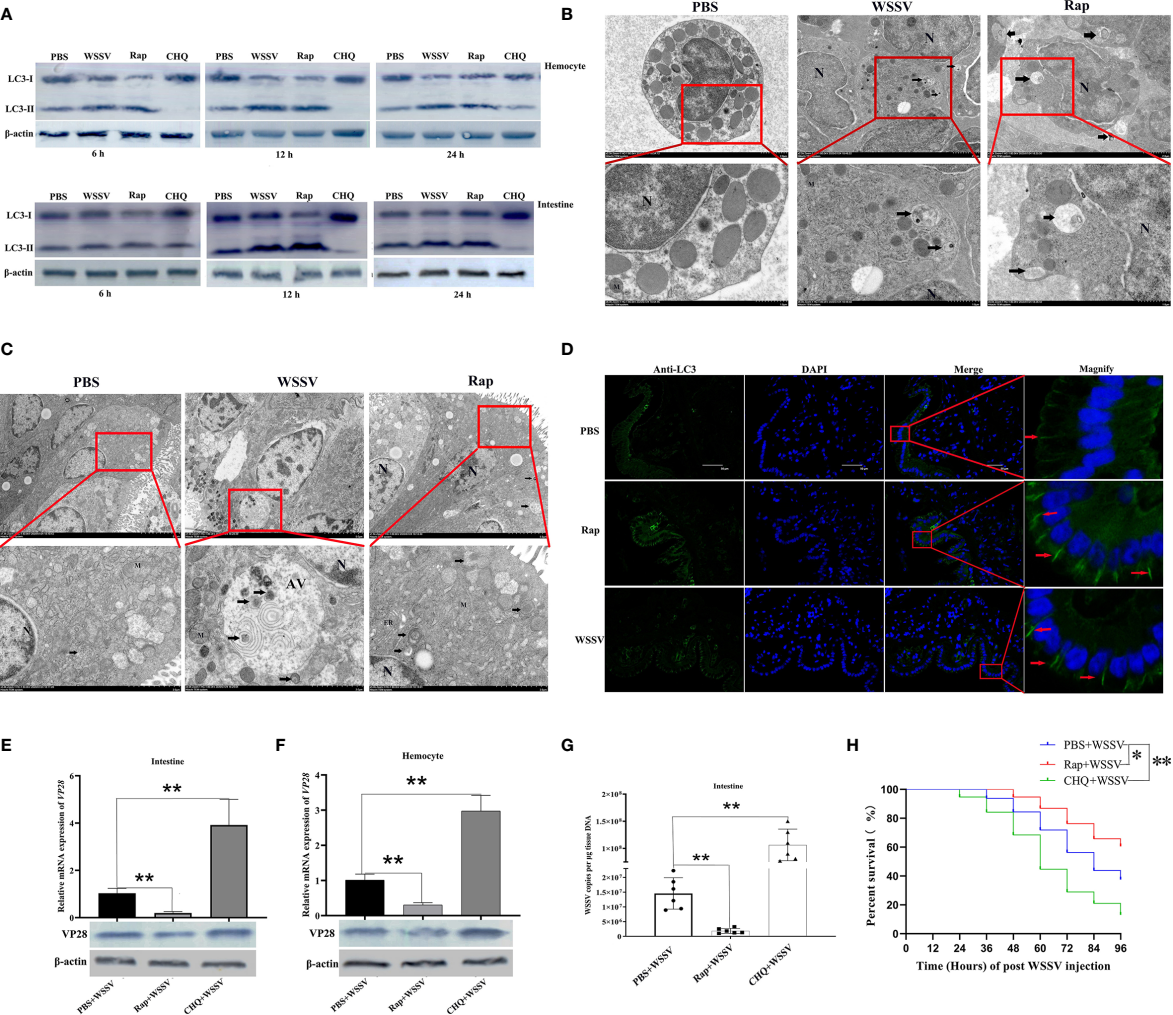
Autophagy played a positive role in restricting WSSV infection in shrimp. **(A)** The expression of LC3-II/LC3-I in hemocytes (upper panel) and the intestine (lower panel) was detected by Western blotting. **(B)** The formation of autophagosome-like vesicles in hemocytes of PBS, WSSV, or Rap challenged shrimp was observed by TEM. Vesicles with the characteristics of autophagosomes are indicated by black arrows. N, nucleus; M, mitochondrion. **(C)** The formation of autophagosome-like vesicles in the intestine of PBS, WSSV, or Rap challenged shrimp was observed by TEM. Vesicles with the characteristics of autophagosomes are indicated by black arrows. N, nucleus; M, mitochondrion; ER, endoplasmic reticulum; AV, autophagic vacuole. **(D)** The distribution of the autophagy marker LC3 in the intestine of PBS, WSSV, or Rap challenged shrimp was detected by immunocytochemistry. Positive immunoreactivities of LC3 are indicated by red arrows. **(E)** The VP28 expression in the intestine of PBS+WSSV group, Rap+WSSV group, and CHQ+WSSV group was determined by using qRT-PCR (upper panel) and western blotting (lower panel). **(F)** VP28 expression in the hemocytes of PBS+WSSV, Rap+WSSV, and CHQ+WSSV groups was determined by using qRT-PCR (upper panel) and western blotting (lower panel). **(G)** Virus copy number in the intestines of PBS+WSSV, Rap+WSSV, and CHQ+WSSV groups was detected by qPCR. **(H)** The survival rates in PBS+WSSV, Rap+WSSV, and CHQ+WSSV groups were recorded. The survival rates were calculated and survival curves were presented as Kaplan-Meier plots. Differences between the two groups were analyzed with log-rank test using the software of GraphPad Prism 8.02. The experiments were repeated at least three times. Asterisks indicate significant differences *P< 0.05 and **P <0.01.

To evaluate whether autophagy occurred in shrimp, rapamycin (Rap, autophagy inducer), chloroquine (CHQ, autophagy inhibitor), and WSSV were injected into shrimp, followed by detection of the expression of LC3-II/LC3-I. The results showed that the expression of LC3-II/LC3-I protein in hemocytes (**Figure 3A**, upper panel) and intestine (**Figure 3A**, lower panel) was increased from 6 h to 24 h after WSSV or Rap challenge (**Figure 3A**). The ratio of LC3-II/LC3-I peaked at 12 h after WSSV or Rap challenge, indicating that autophagy was induced by Rap or WSSV challenge. Conversely, the ratio of LC3-II/LC3-I in CHQ-injected shrimp was lower than that in the control group, which showed that autophagy was inhibited by CHQ challenge in shrimp (**Figure 3A**). To further determine the autophagy level triggered by WSSV infection, we observed autophagosome-like vesicles in WSSV-challenged shrimp of intestines and hemocytes by transmission electron microscopy (TEM). Rap challenge was used as a positive control. In hemocytes, no autophagosome-like vesicles were found in the PBS-challenged shrimp. However, double or single membrane autophagosome-like vesicles (marked by black arrows) were clearly found in the hemocytes of WSSV- and Rap-challenged shrimp (**Figure 3B**). In the intestine, the number of autophagosome-like vesicles in WSSV- and Rap-challenged shrimp was found to be higher than that in PBS-challenged shrimp (**Figure 3C**). Large autophagic vesicles were also observed in the intestine. The immunocytochemistry results showed that the positive immunoreactivity of LC3 in WSSV- and Rap-challenged shrimp was stronger and more concentrated (marked by red arrows) than in the PBS control group (**Figure 3D**). These results indicate that autophagy was induced by WSSV infection in *P. monodon.*

To investigate the relationship between autophagy and WSSV infection, WSSV was injected into shrimp 12 h after injection of Rap or CHQ. WSSV replication was then detected 72 h after WSSV infection. In the intestine and hemocytes, the expression of *VP28* mRNA was decreased significantly in the Rap+WSSV group, which was 0.18-fold and 0.3-fold lower than that in the PBS+WSSV group, respectively (*p<0.01*, **Figures 3E, F**, upper panel). The expression of *VP28* mRNA was increased significantly in the intestine and hemocytes of the CHQ+WSSV group, which was 3.8-fold and 2.9-fold higher than that in the PBS+WSSV group, respectively (*p<0.01*, **Figures 3E, F**, upper panel). Western blotting results showed that the expression of VP28 protein in the intestine and hemocytes was decreased in the Rap+WSSV group and increased in the CHQ+WSSV group, compared with the PBS+WSSV group (**Figures 3E, F**, lower panel). In the intestine, the copy number of WSSV in the Rap+WSSV group (1.8 × 10^6^ copies copies per μg genomic DNA) was significantly lower than that in the PBS+WSSV group (1.4 × 10^7^ copies per μg genomic DNA) (*p<0.01*, **Figure 3G**). However, the copy number of WSSV in the CHQ+WSSV group (1×10^8^ copies per μg genomic DNA) was significantly higher than that in the PBS+WSSV group (*p<0.01*, **Figure 3G**). In addition, the survival rates in the Rap+WSSV and CHQ+WSSV groups were 60.5% and 13.1%, respectively, which were significantly higher and lower than those in the PBS+WSSV group (survival rate 37.5%) (**Figure 3H**). These data suggest that host autophagy plays a positive role in restricting WSSV replication in shrimp.

### PmTRIM50-Like Was Required for Autophagy in Shrimp

**Figure 4:**
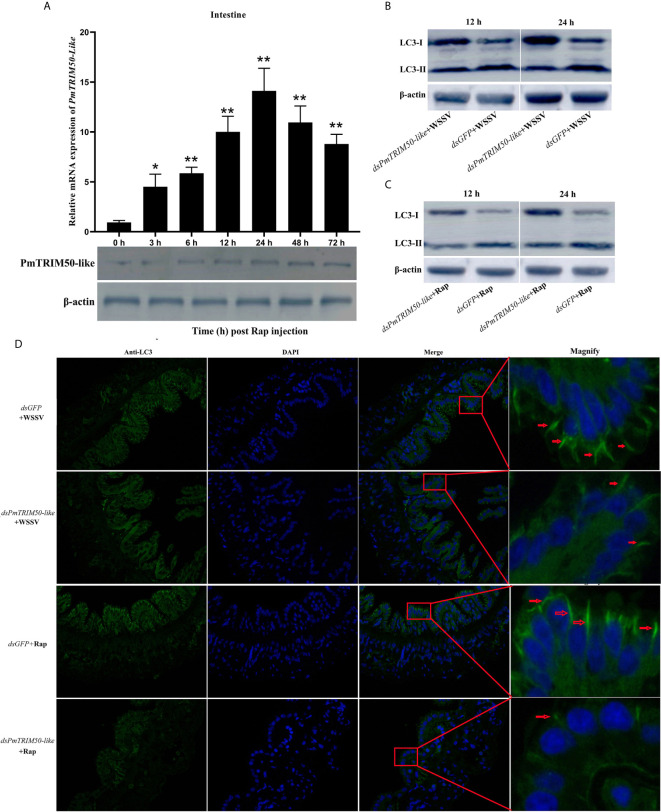
PmTRIM50-like was required for autophagy in shrimp. **(A)** Expression patterns of PmTRIM50-like mRNA and protein were detected by qRT-PCR (upper panel) and western blotting (lower panel) in the intestine after rapamycin challenge. *β-actin* was used as an internal reference. **(B)** The expression of LC3-II/LC3-I in *dsPmTRIM50-like*+WSSV and *dsGFP*+WSSV groups was detected by western blotting. **(C)** The expression of LC3-II/LC3-I in *dsPmTRIM50-like*+Rap and *dsGFP*+Rap groups was detected by western blotting. **(D)** The distribution of the autophagy marker LC3 in *dsPmTRIM50-like*+WSSV, *dsGFP*+WSSV, *dsPmTRIM50-like*+Rap, and *dsGFP*+Rap groups was detected by immunocytochemistry. Positive immunoreactivities of LC3 are indicated by red arrows. Asterisks indicate significant differences (*P<0.05 and **P<0.01) compared with values in the control group. Error bars represent mean ± S.D. of three independent PCR amplifications and quantifications.

Many TRIM proteins are critical components and regulators of the autophagy machinery. To investigate whether PmTRIM50-like is involved in autophagy in shrimp, the expression of PmTRIM50-like was detected after Rap challenge. The results showed that *PmTRIM50-like* was significantly upregulated (from 4.5- to 14-fold) in the intestine after Rap challenge (**Figure 4A**, upper panel). Western blotting analysis revealed that the protein expression level of PmTRIM50-like was also increased in the intestine after Rap challenge (Fig. 4A, lower panel). The positive correlation between the expression of PmTRIM50-like and autophagy suggested that PmTRIM50-like might be involved in autophagy in shrimp.

In mammals, some TRIMs can interact with cargo/target-recognizing proteins such as p62 and core regulators of autophagy and form protein complexes called ‘TRIMosomes’ (18). To investigate the role of PmTRIM50-like in autophagy in shrimp, shrimp were treated with *dsPmTRIM5-like* or *dsGFP*, followed by WSSV or Rap. Autophagy was detected by western blotting and immunocytochemistry. Compared with the *dsGFP*+WSSV group, the ratio of LC3-II/LC3-I decreased in the *dsPmTRIM50-like*+WSSV group (**Figure 4B**). The immunocytochemistry results showed that the positive immunoreactivity of LC3 in the *dsPmTRIM50-like*+WSSV group was weaker and fewer than in the *dsGFP*+WSSV group (**Figure 4D**), indicating that PmTRIM50-like positively regulates WSSV-induced autophagy. Compared with the d*sGFP*+Rap group, the levels of autophagy were also decreased in the *dsPmTRIM50-like*+Rap group as detected by western blotting (**Figure 4C**) and immunocytochemistry (**Figure 4D**), indicating that PmTRIM50-like positively regulates Rap-induced autophagy. These results showed that host autophagy was inhibited when the expression of PmTRIM50-like was silenced in shrimp. This strongly suggests that PmTRIM50-like might be required for autophagy induction in shrimp.

### PmTRIM50-Like Restricted WSSV Infection *via* Mediating Autophagy in Shrimp

**Figure 5:**
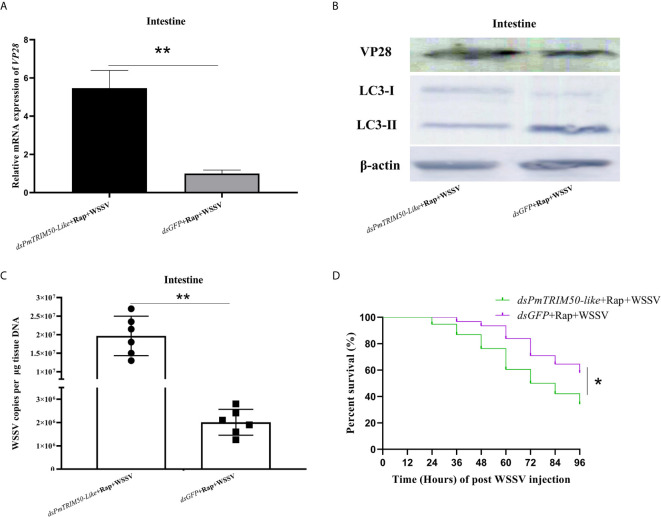
PmTRIM50-like restricted WSSV infection *via* mediating autophagy in shrimp. **(A)** The expression of VP28 in *dsPmTRIM50-like*+Rap+WSSV and *dsGFP*+Rap+WSSV groups was detected by qRT-PCR. **(B)** The expressions of VP28 protein (upper panel) and LC3-II/LC3-I (middle panel) in *dsPmTRIM50-like*+Rap+WSSV and *dsGFP*+Rap+WSSV groups were detected by western blotting. *β-actin* was used as control (lower panel). **(C)** Virus copy number of WSSV in the intestines of *dsPmTRIM50-like*+Rap+WSSV and *dsGFP*+Rap+WSSV groups were detected by qPCR. **(D)** The survival rates in *dsPmTRIM50-like*+Rap+WSSV and *dsGFP*+Rap+WSSV groups were recorded. The survival rates were calculated and survival curves were presented as Kaplan-Meier plots. Differences between the two groups were analyzed with log-rank test using the software of GraphPad Prism 8.02. The experiments were repeated three times. Asterisks indicate significant differences *P< 0.05 and **P <0.01.

One mechanism of action of TRIM proteins in antiviral autophagy is to regulate autophagy activity during viral infection (18). In this study, autophagy could restrict WSSV replication, while PmTRIM50-like could both promote autophagy and restrict WSSV replication in shrimp. This indicated that PmTRIM50-like might restrict WSSV replication by mediating autophagy. To investigate whether PmTRIM50-like regulated anti-WSSV autophagy, shrimp were co-injected with dsPmTRIM50-like and Rap, followed by WSSV challenge. Host autophagy and WSSV replication were also detected. The results showed that the expression of LC3-II/LC3-I in the *dsPmTRIM50-like*+Rap+WSSV group was lower than that in the *dsGFP*+Rap+WSSV group (**Figure 5B**, middle panel). The replication of WSSV in the d*sPmTRIM50-like*+Rap+WSSV group increased significantly compared with the *dsGFP*+Rap+WSSV group, as a higher VP28 expression (**Figures 5A, B**, upper panel), higher WSSV copies (**Figure 5C**), and lower survival rate (**Figure 5D**) were detected in the *dsPmTRIM50-like*+Rap+WSSV group. These results further showed that PmTRIM50-like is indispensable for autophagy induction and thus, a possible mechanism for PmTRIM50-like-mediated inhibition of WSSV replication may be *via* mediating autophagy in shrimp.

### PmTRIM50-Like Recognized Components of WSSV and Targeted Them for Ubiquitination *In Vitro*

**Figure 6:**
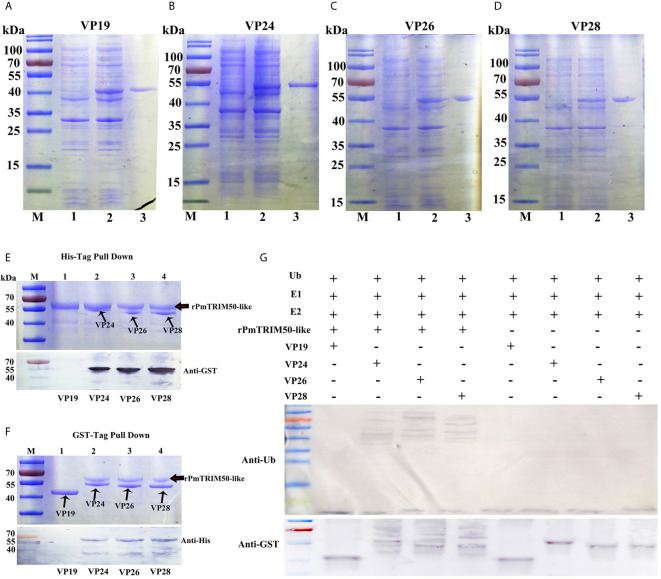
PmTRIM50-like recognized components of WSSV and targeted them for ubiquitination *in vitro*. **(A–D)** Purified GST tagged WSSV structural proteins of VP19 **(A)**, VP24 **(B)**, VP26 **(C)**, and VP28 **(D)**. **(E)** His tagged PmTRIM50-like that interacted with GST-VP19, VP24, VP26, and VP28 was obtained in His pull-down assay and visualized by coomassie blue staining (upper panel) and confirmed by western blotting with anti-GST antibody (lower panel). **(F)** GST tagged VP19, VP24, VP26, and VP28 that interacted with His-PmTRIM50-like was obtained in GST pull-down assay and visualized by coomassie blue staining (upper panel) and confirmed by western blotting with anti-His antibody (lower panel). **(G)** In vitro ubiquitination of VP19, VP24, VP26, and VP28 by rPmTRIM50-like plus E1, E2, ATP and ubiquitin (Ub) as detected by anti-Ub antibody (upper panel) and anti-GST antibody (lower panel).

Another mechanism of action of TRIM proteins acting in antiviral autophagy is to act as specific cargo receptors that directly recognize viral components and target them for degradation by autophagy (18). To investigate the possibility of PmTRIM50-like to recognize WSSV components, we conducted GST pulldown and *in vitro* ubiquitination assays. First, the GST-tagged proteins VP19, VP24, VP26, and VP28 were expressed and purified in *E. coli* BL21 cells (**Figures 6A–D**). The pulldown assay showed that interaction occurred between rPmTRIM50-like and three viral envelope proteins VP24, VP26, and VP28. However, no interaction was detected between rPmTRIM50-Like and VP19 (**Figures 6E, F**). Many TRIM proteins contain a typical RING-finger domain and have E3 ubiquitin ligase activity, and thus, can directly couple ubiquitin to a specific substrate. The *in vitro* ubiquitination assay showed that PmTRIM50-like could ubiquitinate WSSV components as an obvious polyubiquitination smear of VP24, VP26, and VP28 were detected by anti-Ub antibody in the presence of rPmTRIM50-Like protein (**Figure 6G**, upper panel). VP24, VP26, and VP28 were also detected by anti-GST antibody with increased molecular weight (**Figure 6G**, lower panel). However, no ubiquitination of VP19 was observed in the presence of rPmTRIM50-like. These results indicated that PmTRIM50-like protein could interact with the viral envelope proteins of WSSV (VP24, VP26, and VP28) and target them for ubiquitination *in vitro*. PmTRIM50-like might act as a cargo receptor that directly recognizes envelope proteins of WSSV and target them for degradation by autophagy in shrimp. Therefore, we concluded that another possible mechanism for PmTRIM50-like-mediated inhibition of WSSV replication might be by recognizing WSSV components and targeting them for degradation.

## Discussion

TRIM proteins are widely recognized as important antiviral restriction factors or modulators of signaling pathways that lead to the induction of antiviral or proinflammatory cytokines. In mammals, TRIM proteins function as autophagy receptors and regulators of autophagosome formation. Many mammalian TRIM proteins play essential roles in viral-induced autophagy. However, there has been no report on the involvement of TRIM proteins in viral infection and host autophagy in crustaceans. In this study, we identified a member of the tripartite motif family proteins (*PmTRIM50-like*) from *P. monodon*, which could restrict WSSV replication by positively regulating autophagy in shrimp. To our knowledge, this is the first report of a TRIM protein being involved in viral infection and host autophagy in crustaceans.

The TRIM family of proteins participate in a variety of biological processes in vertebrates, including playing an important role in the innate immune response and virus-induced autophagy (18). Research has shown that roughly half of the 75 human TRIM family proteins can enhance the innate immune response (42). Due to their importance in regulating the innate immune response, TRIMs have become an increasingly studied protein family. Typically, TRIM proteins are composed of a RING finger domain, one or two B-BOX domains, a coiled-coil region in the N-terminus, and various specific domains in the C-terminus, although eight TRIM proteins in humans lack the RING finger domain (43). Many proteins containing the RING domain have E3 ubiquitin ligase activity and can activate specific signaling pathways by targeting specific proteins for degradation, thereby regulating the antiviral immune response (44). For example, the RING finger protein CqRNF152-like in *Cherax quadricarinatus* has self-ubiquitination activity and interacts with WSSV VP28 (45). Porcine RING finger protein 114 inhibits classical swine fever virus replication *via* K27-linked polyubiquitination of viral NS4B (46). The PmTRIM50-like protein contains a typical RING domain, a B-box domain, and a cyclophilin domain in the C terminus, indicating that PmTRIM50-like might contain E3 ubiquitin ligase activity and have antiviral function. The phylogenetic tree showed that PmTRIM50-like was most closely related to *Talpa occidentalis* E3 ubiquitin-protein ligase TRIM50 and hence was named PmTRIM50-like in this study. Upon WSSV challenge, the expression of PmTRIM50-like was upregulated in both hemocytes and intestines, suggesting that PmTRIM50-like was responsive to WSSV infection. Among TRIM family proteins, many TRIM proteins are widely recognized as important antiviral restriction factors or modulators of signaling pathways (47). In this study, shrimps with knockdown of *PmTRIM50-like* were more susceptible to WSSV infection as higher virus copies and higher mortality rates were detected. However, shrimps injected with *PmTRIM50-like* mRNA were more resistant to WSSV infection and lower virus copies were detected, suggesting that PmTRIM50-like restricted WSSV replication in *P. monodon.*

Autophagy is a homeostatic process that not only sustains cell survival under stress but is also induced during a viral infection (48). Considering the important role of autophagy in antiviral processes, some viruses are equipped with sophisticated mechanisms to modulate autophagy in order to facilitate the stability and replication of the virus (49–51). On one hand, autophagy can be induced by a few viruses (14). On the other hand, autophagy can be inhibited and subverted by a few viruses to promote their replication (52). Previous studies have shown that autophagy can be induced by WSSV infection in *Marsupenaeus japonicus* and *Cherax quadricarinatus* (53, 54). In this study, the ratio of LC3-II/LC3-I protein was upregulated by WSSV challenge. In the TEM observation of WSSV-infected shrimp, we observed an increased number of autophagosome-like vesicles and autophagic vesicles in hemocytes and intestines. The LC3 positive signal in WSSV-challenged shrimp was stronger and more concentrated than that in the PBS control group. These results indicate that WSSV infection promotes autophagy in *P. monodon*. As mentioned earlier, the impact of host autophagy on viral replication is highly virus-and cell-type-specific. Autophagy can regulate innate and acquired immunity to protect against viral infection (12). However, autophagy can also facilitate viral replication by serving as a site for viral replication (55). In our study, the amount of WSSV decreased significantly in shrimp after the induction of autophagy by Rap, compared with the control. The amount of WSSV increased significantly in shrimp after the inhibition of autophagy by chloroquine. These results were similar to those of previous studies in which Rap-induced autophagy played a role in restricting WSSV replication in hematopoietic tissue stem cells of *Cherax quadricarinatus* (54). Chloroquine inhibits autophagic flux by decreasing autophagosome-lysosome fusion. In *Marsupenaeus japonicus*, the amount of WSSV increased significantly when kuruma shrimp were challenged with chloroquine, indicating that inhibiting autophagy increased the amount of WSSV in shrimp (31). Our results indicate that host autophagy is probably involved in the clearance of WSSV in *P. monodon*.

Some TRIM proteins are critical components or regulators of autophagy machinery. For example, TRIM5a can interact with LC3s/GABARAPs, p62, and Beclin1 to form a core complex of the autophagy machinery (21). TRIM13 localizes to the ER and induces autophagy during ER stress *via* its coiled-coil domain and interacts with p62 (56). TRIM59 regulates autophagy by modulating the transcription and ubiquitination of Beclin1 (57). In this study, host autophagy was found to be induced by WSSV and rapamycin challenge in shrimp. The expression of PmTRIM50-like was upregulated by WSSV and rapamycin challenge. With the increase in WSSV- or rapamycin-induced autophagy, the expression level of PmTRIM50-like also increased, indicating that PmTRIM50-like might be involved in the process of autophagy in shrimp. However, WSSV- or rapamycin-induced autophagy could be clearly damaged by silencing of PmTRIM50-like in shrimp, indicating that PmTRIM50-like might be required for autophagy induction in shrimp.

TRIM family proteins can act as antiviral autophagy inducers in two different ways. A few TRIM proteins are critical components of the autophagy machinery that mediate viral clearance. TRIM23 is a core component of the autophagy machinery and is required for the induction of antiviral autophagy by several viruses (24). TRIM16 was found to promote antiviral autophagy by facilitating activation of the p62-NRF2 axis (58). In this study, rapamycin-induced autophagy significantly restricted the replication of WSSV, but when *PmTRIM50-like* was silenced, rapamycin-induced autophagy was inhibited, resulting in an increase in WSSV replication. This demonstrated that PmTRIM50-like might restrict WSSV replication by mediating autophagy.

Certain TRIMs can act as specific cargo receptors that directly recognize viral components and target them for degradation by autophagy. For example, TRIM5a directly interacts with the capsid of human immunodeficiency virus-1 (HIV-1), leading to premature disassembly of the capsid (59). The recognition of HIV-1 capsid protein (p24) by TRIM5a leads to proteasomal and autophagosome degradation. For autophagic clearance, the p24-TRIM5a complex is recruited to autophagosomes, where TRIM5a induces autophagy in a Beclin-1- and ULK1-dependent manner (59). The recognition of viral components by other TRIM proteins may also destroy virus replication in different ways. TRIM11 directly interacts with the capsid of human immunodeficiency virus-1 (HIV-1) to affect the release of HIV-1 particles (60). TRIM25 targets CpG-rich sites in the genomes of Sindbis virus (SINV) and HIV-1, and inhibits translation of the incoming virus genome (61, 62). TRIM21 suppresses hepatitis B virus (HBV) DNA replication by promoting the ubiquitination of HBV DNA polymerase (63). TRIM41 targets the viral nucleoprotein of vesicular stomatitis virus (VSV) for ubiquitination and subsequent protein degradation (64). The recognition of viral components can directly lead to premature capsid disassembly and degradation, block the lifecycle of the virus, or act as “autophagy receptors” to initiate autophagy. In this study, PmTRIM50-like could interact with VP24, VP26, and VP28, and target them for ubiquitination *in vitro*. VP19, VP24, VP26, and VP28 are the four major envelope proteins of WSSV and function in virus entry and systemic infection (65–67). VP24, VP26, and VP28 share high sequence homology with each other; however, VP19 has a unique structure and biological character (68, 69). Therefore, we concluded that one possible mechanism for PmTRIM50-like to restrict WSSV replication was by recognizing envelope proteins of WSSV and targeting them for ubiquitination. The recognition and ubiquitination of WSSV might lead to the disassembly and degradation of WSSV by autophagy or proteasomes in shrimp.

In summary, knockdown and overexpression analyses revealed that PmTRIM50-like possessed antiviral functions in shrimp. Further, our study found that PmTRIM50-like was required for autophagy induction and could restrict WSSV replication by mediating autophagy. Moreover, we discovered that PmTRIM50-like could interact with WSSV envelope proteins and target them for ubiquitination *in vitro*.

**Figure 7:**
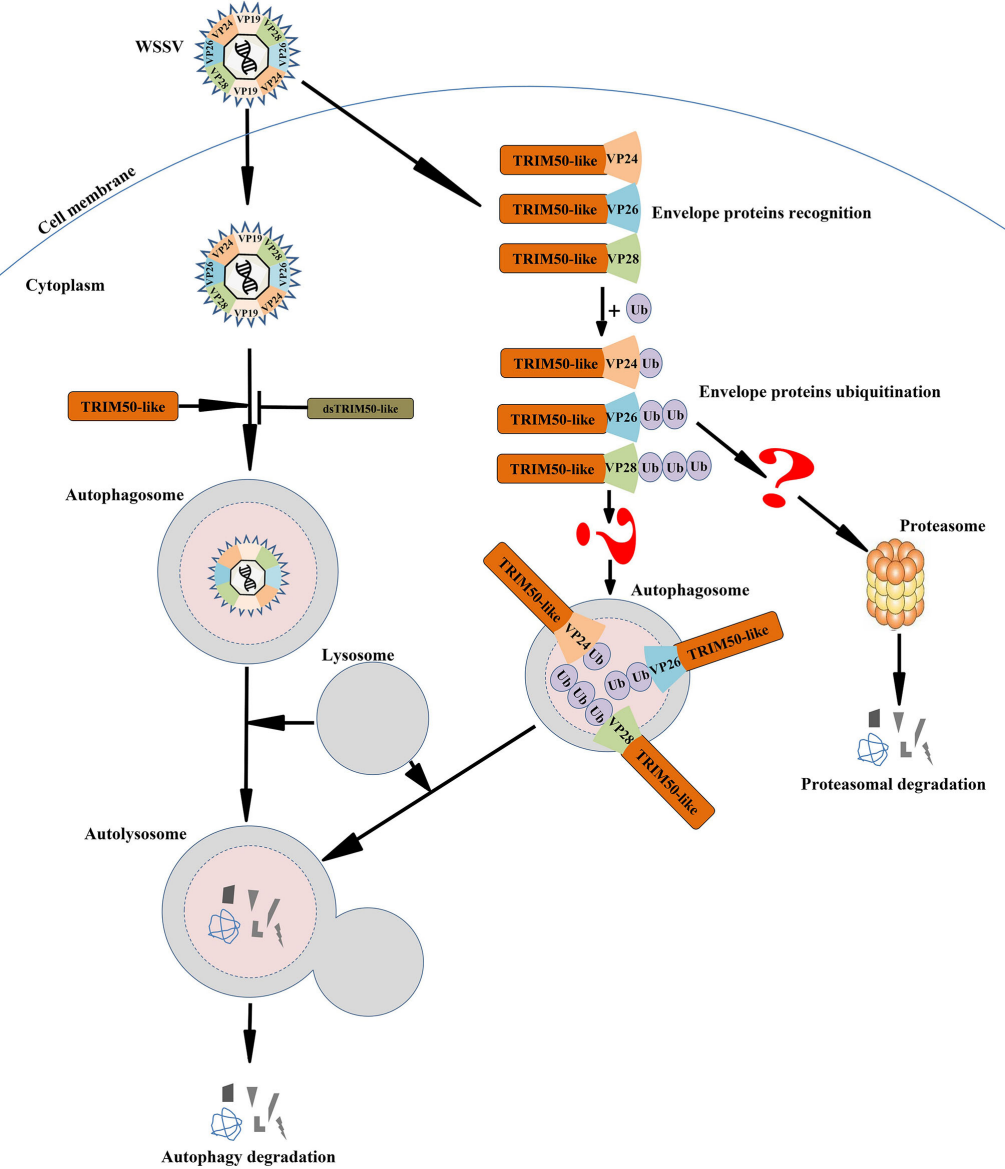
Predicted schematic diagram for PmTRIM50-like-mediated anti-WSSV autophagy in shrimp. After WSSV infection, the expression of PmTRIM50-like was upregulated and autophagy was induced in shrimp. Subsequently, autophagy degraded WSSV in a PmTRIM50-like-dependent manner. In shrimp cells, PmTRIM50-like might act as a cargo receptor to recognize and ubiquitinate WSSV envelope proteins of VP24, VP26, and VP28. The recognition and ubiquitination might mark and target WSSV for degradation by autophagy.

In conclusion, PmTRIM50-like mediates anti-WSSV autophagy in *P. monodon.* After WSSV infection, the expression of PmTRIM50-like was upregulated, and autophagy was induced in shrimp. Subsequently, autophagy degraded WSSV in a PmTRIM50-like-dependent manner. In shrimp cells, PmTRIM50-like may act as a cargo receptor to recognize and ubiquitinate the WSSV envelope proteins VP24, VP26, and VP28. Moreover, recognition and ubiquitination might mark and target WSSV for degradation by autophagy (**Figure 7**). This is the first report of a TRIM family protein mediating antiviral autophagy in shrimp. However, the molecular mechanism by which PmTRIM50-Like regulates antiviral autophagy in shrimp needs further investigation.

## Data Availability Statement

The original contributions presented in the study are included in the article/**Supplementary Material**. Further inquiries can be directed to the corresponding author.

## Author Contributions

CZ and CP performed the experiments and written original draft preparation. CZ: reviewed and edited the paper. PW, LY, and SF: provided resources. LQ: acquired the funding. All authors contributed to the article and approved the submitted version.

## Funding

This study was supported by National Key R&D Program of China (2018YFD0900103); Central Public-interest Scientific Institution Basal Research Fund, CAFS (2020TD21); National Natural Science Foundation of China (42006113); and the Central Public-interest Scientific Institution Basal Research Fund, South China Sea Fisheries Research Institute, CAFS (2019TS11).

## Conflict of Interest

The authors declare that the research was conducted in the absence of any commercial or financial relationships that could be construed as a potential conflict of interest.
