# Supplementary material for: Identification of a Shrimp E3 Ubiquitin Ligase TRIM50-Like Involved in Restricting White Spot Syndrome Virus Proliferation by Its Mediated Autophagy and Ubiquitination
Source: Front Immunol. 2021 May 11;12:682562. doi: 10.3389/fimmu.2021.682562 (PMC8144704; doi:10.3389/fimmu.2021.682562)
Supplement: Supplementary file 1 [file DataSheet_1.pdf]

Supplementary Figures

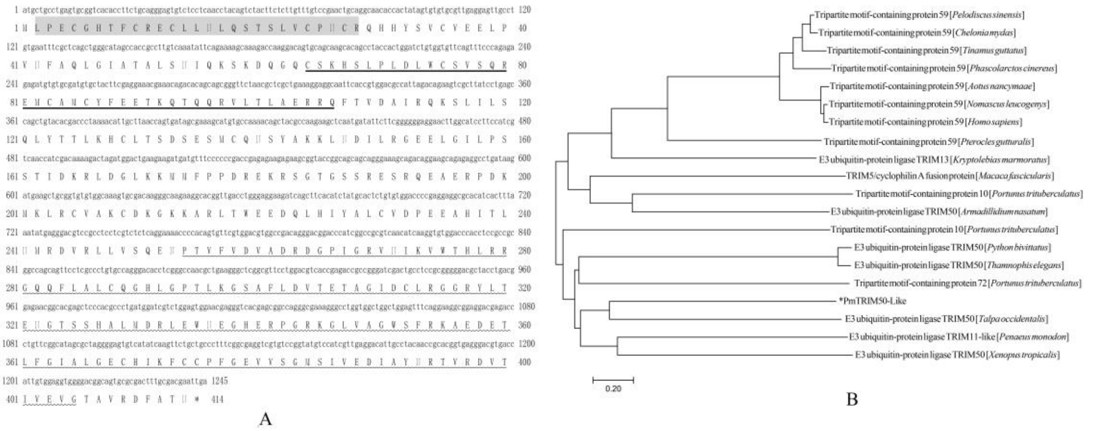

Supplementary Figure 1. Molecular characterization of *PmTRIM50-like*. (A) The nucleotide and deduced amino acid characteristic of *PmTRIM50-like*; Gray region was RING domain; B-Box domains were bolded underlined. Cyclophilin super family domain was marked in wavy line. (B) Phylogenetic analyses of *PmTRIM50-like* (black asterisk) and other homologous genes from other species (XP_037788838.1, E3 ubiquitin-protein ligase TRIM11-like [*Penaeus monodon*]; MPC09098.1, Tripartite motif-containing protein 10 [*Portunus trituberculatus*]; MPC27339.1, Tripartite motif-containing protein 10 [*Portunus trituberculatus*]; MPC09100.1, Tripartite motif-containing protein 72 *[Portunus trituberculatus*]; KAB7494620.1, E3 ubiquitin-protein ligase TRIM50 [*Armadillidium nasatum*]; ACU46018.1, TRIM5/cyclophilin A fusion protein [*Macaca fascicularis*]; KFU97184.1, Tripartite motif-containing protein 59, partial [*Pterocles gutturalis*]; XP_006123500.1, tripartite motif-containing protein 59 [*Pelodiscus sinensis*]; XP_007441599.1, E3 ubiquitin-protein ligase TRIM50 [*Python bivittatus*]; XP_012302766.1, tripartite motif-containing protein 59 [*Aotus nancymaae*]; XP_003256425.1, tripartite motif-containing protein 59 [*Nomascus leucogenys*]; XP_017949750.1, E3 ubiquitin-protein ligase TRIM50 [*Xenopus tropicalis*]; XP_020844498.1, tripartite motif-containing protein 59 [*Phascolarctos cinereus*]; XP_017265762.1, E3 ubiquitin-protein ligase TRIM13 [*Kryptolebias marmoratus*]; EMP32723.1, Tripartite motif-containing protein 59, partial [*Chelonia mydas*]; XP_032071003.1, E3 ubiquitin-protein ligase TRIM50 [*Thamnophis elegans*]; KGL84188.1, Tripartite motif-containing protein 59, partial [*Tinamus guttatus*]; XP_037362732.1, E3 ubiquitin-protein ligase TRIM50 [*Talpa occidentalis*]; NP_775107.1, tripartite motif-containing protein 59 [*Homo sapiens*]) .
